# Evaluation of Cognitive Behavioral Therapy on Improving Pain, Fear Avoidance, and Self-Efficacy in Patients with Chronic Low Back Pain: A Systematic Review and Meta-Analysis

**DOI:** 10.1155/2022/4276175

**Published:** 2022-03-19

**Authors:** Jiajia Yang, Wai Leung Ambrose Lo, Fuming Zheng, Xue Cheng, Qiuhua Yu, Chuhuai Wang

**Affiliations:** ^1^Department of Rehabilitation Medicine, The First Affiliated Hospital, Sun Yat-Sen University, Guangzhou, China; ^2^Guangdong Engineering and Technology Research Center for Rehabilitation Medicine and Translation, The First Affiliated Hospital, Sun Yat-Sen University, Guangzhou, China

## Abstract

**Background:**

Cognitive-behavioral therapy (CBT) is commonly adopted in pain management programs for patients with chronic low back pain (CLBP). However, the benefits of CBT are still unclear.

**Objectives:**

This review investigated the effectiveness of CBT on pain, disability, fear avoidance, and self-efficacy in patients with CLBP.

**Methods:**

Databases including PubMed, EMBASE, Web of Science, Cochrane Library, and PsycINFO were searched. RCTs examining the effects of CBT in adults with CLBP were included. The data about the outcome of pain, disability, fear avoidance, and self-efficacy were retained. Subgroup analysis about the effects of CBT on posttreatment was conducted according to CBT versus control groups (waiting list/usual care, active therapy) and concurrent CBT versus CBT alone. A random-effects model was used, and statistical heterogeneity was explored.

**Results:**

22 articles were included. The results indicated that CBT was superior to other therapies in improving disability (SMD −0.44, 95% CI −0.71 to −0.17, *P* < 0.05), pain (SMD −0.32, 95% CI −0.57 to −0.06, *P* < 0.05), fear avoidance (SMD −1.24, 95% CI −2.25 to −0.23, *P* < 0.05), and self-efficacy (SMD 0.27, 95% CI 0.15 to 0.40, *P* < 0.05) after intervention. No different effect was observed between CBT and other therapies in all the follow-up terms. Subgroup analysis suggested that CBT in conjunction with other interventions was in favor of other interventions alone to reduce pain and disability (*P* < 0.05).

**Conclusion:**

CBT is beneficial in patients with CLBP for improving pain, disability, fear avoidance, and self-efficacy in CLBP patients. Further study is recommended to investigate the long-term benefits of CBT. This meta-analysis is registered with Prospero (registration number CRD42021224837).

## 1. Introduction

Low back pain (LBP) is a public health concern that contributes to years lived with disability globally [1]. Globally, the estimated age-standardized point prevalence of LBP was 7.50% in 2017 [2] and ranked ninth for the cause of years lived with disability and health burden [3]. One study from the UK in 2018 claimed that about 10 to 15% of LBP cases go on to develop chronic low back pain (CLBP) which is defined as pain lasting over 12 weeks [4]. A review published in 2015 based on cross-sectional population and cohort studies reported that the CLBP prevalence was 19.6% in those aged between 20 and 59 [5]. CLBP is considered to have multifaceted pathophysiology that is influenced by somatic pathology and psychological and social factors [6]. Psychological indicators such as depression, anxiety, fear avoidance, and low self-efficacy are associated with an increased risk of developing pain and disability in patients with CLBP [7–10]. The management of CLBP includes medication and therapeutic exercise. While these interventions demonstrated modest improvement, pain recurrence remains [11]. One guideline in 2017 recommended a combined psychological and physical approach if previous treatment was ineffective or in cases where medium to high risk of chronicity was identified [12].

Cognitive-behavioral therapy (CBT) is a set of interventions that involve 4 broad components: the patient's knowledge and understanding about pain and their pain perception, the learning of active coping strategies, maintaining the coping strategies, and problem-solving plans to deal with pain and challenging situation. Although there are several systemic reviews of CBT intervention for pain alleviation in CLBP patients, most reviews have limitations. One is the restriction of a single comparison group [13]. Two reviews did not evaluate the long-term effects of CBT on CLBP patients [13, 14]. Two systemic reviews investigated the long-term effects but were published in the years 2007 and 2015 where their findings may be out-of-date [15, 16]. Therefore, the clinical benefits of CBT to reduce pain and disability immediately after treatment and during follow-up periods remain unclear. An updated pooled estimation of quantitative analysis with a larger sample size than previous studies would provide adequate power to evaluate the posttreatment and long-term effects of CBT in CLBP patients.

Psychological factors such as depression, anxiety, fear avoidance, and low self-efficacy are related to increased risk in patients with CLBP [7–10]. However, there has been already one review that investigated the effects of CBT on depression reduction in patients with CLBP [13]. In addition, there are not enough articles evaluating anxiety to complete a review. Therefore, the psychological outcomes in the present study were fear avoidance and self-efficacy instead of depression and anxiety. Touche et al. made a cross-sectional study to investigate the relationship between psychological variables, lumbar spine range of motion, and pain intensity in patients with CLBP. The results indicated that patients with low self-efficacy tend to increase pain intensity during lifting tasks [17]. Fear avoidance is characterized by escape and avoidance behaviors. The immediate consequences are reduced participation in daily activities due to the expectation of pain exacerbation [18]. For patients with CLBP, longstanding avoidance and physical inactivity have a detrimental impact on the musculoskeletal and cardiovascular systems, leading to disuse syndrome that further worsens the pain problem [19]. Therefore, specific intervention strategies should be implemented to improve self-efficacy and fear avoidance in patients with CLBP to achieve a positive clinical outcome. Wenzel et al. claimed that CBT may modify maladaptive behaviors and overcome avoidance behavior to improve self-care [20]. However, there is still a lack of review about the effects of CBT on improving self-efficacy and fear avoidance for CLBP patients. Therefore, it is imperative to conduct a systemic review to assess (1) the benefits of CBT on pain and disability relief at posttreatment and during different follow-up periods and (2) the effectiveness of CBT on improving fear avoidance and self-efficacy in patients with CLBP.

## 2. Materials and Methods

This review followed the guidelines for Preferred Reporting Items for Systematic reviews and Meta-analyses (PRISMA) (see Supplementary Material 1 for detailed information of PRISMA Checklist). The protocol was registered with Prospero (registration number CRD42021224837).

### 2.1. Criteria for Considering Studies for This Review

Studies were included in the review if they were randomized controlled trials (RCTs) that evaluated the effects of CBT on patients diagnosed with CLBP.

The inclusion criteria were as follows: (1) patients (>18 years old) diagnosed with chronic low back pain (pain duration >3 months); (2) with or without leg pain; (3) studies adopted CBT alone or CBT combined with other therapies as an intervention arm; (4) CBT delivered face-to-face, web-based, or telephone-based in one to one or group-based setting; (5) the comparison arm that may include waiting list (WL), usual care (UC), or any other active therapies (AT): exercise, physical therapy, or drug therapy; and (6) acceptance and commitment therapy.

Exclusion criteria were as follows: (1) patient population having other specific pathology, including spinal stenosis, lumbar instability, postsurgical pain, pregnancy-related LBP, spinal fractures, cauda equina, or spinal tumors; (2) other chronic pain caused by other pathologies such as rheumatoid arthritis, polymyalgia rheumatic, and fibromyalgia; and (3) lack of documentation of the CBT content.

### 2.2. Primary and Secondary Outcomes

Pain intensity and disability level were the primary outcomes. Fear of avoidance and/or self-efficacy were the secondary outcomes. Pain intensity was evaluated by the Visual Analog Scale (VAS) or Numeric Rating Scale (NRS). Disability was measured by the Roland Morris Disability Questionnaire (RMDQ) or the Oswestry Disability Index (ODI) and Activities of Daily Living (ADL). Fear of avoidance was evaluated by the Fear-Avoidance Beliefs Questionnaire (FABQ). Self-efficacy was assessed by the Pain Self-efficacy Questionnaire (PSEQ).

### 2.3. Search Methods for Identification of Studies and Data Extracted

Articles were searched in the following five electronic databases: (1) PubMed, (2) EMBASE, (3) Web of Science, (4) Cochrane Library, and (5) PsycINFO. All literature studies published in English between 1^st^ January 1980 and 20^th^ November 2021 were searched without any restriction of countries. Reference lists of all selected articles were independently screened to identify additional studies that were not identified in the initial search. Two reviewers (J. Y., F. Z) screened for eligible studies from titles and abstracts. Potentially relevant studies were obtained in full text and independently assessed for inclusion. Two reviewers (J. Y., F. Z) extracted data and assessed the quality of the evidence independently. Two reviewers (J. Y., X. C) assessed the risk of bias independently. Any disagreement was discussed to reach a consensus during moderation meetings.

The following data were extracted using a standardized format: author's name, year of publication, the country where the study was conducted, study period, follow-up time, the total number of people included in the study, blinding types, randomization procedure, and sociodemographic characteristics. The outcome of pain intensity, disability, fear of avoidance, and self-efficacy were recorded before and after the intervention and at the follow-up time points of 3, 6, and 12 months. Outcome values were extracted or converted into mean and standard deviations. If there were missing values, the authors attempted to contact the authors to acquire the missed values.

### 2.4. Assessment of Risk of Bias in Included Studies

The risk of bias for each study was independently assessed by two reviewers according to the 13 criteria recommended by the Cochrane Back and Neck Review Group [21]. It is a tool that is the same as the recommended Cochrane Collaboration but has additional items relevant to the assessment of nondrug trials. It also contains 6 domains of selection bias, performance bias, detection bias, attritions bias, reporting bias, and other bias. For each study, each criterion was scored as “low,” “high,” or “unclear risk.” A consensus method was adopted to conclude the risk of bias of the included studies. However, if an agreement was not achieved at any stage, a third review author was consulted. The GRADE (Grading of Recommendations Assessment, Development, and Evaluation) guideline was applied to assess the confidence of the effect estimates based on the following criteria: risk of bias, inconsistency, imprecision, indirectness, and publication bias.

### 2.5. Measures of Treatment Effects

Data analysis was conducted in Review Manager Software 5.4.1. All of the outcomes were performed as the change values of mean difference and standard deviation (SD) before treatment and after treatment. As these outcomes were continuous data from different scales, the effect sizes were calculated by standardized mean difference (SMD) and 95% confidence intervals. A two-sided *P* value of less than 0.05 was considered statistically significant. A random-effects model was used to analyze the data. A negative effect size indicated that CBT was more beneficial than the comparison therapies. When there were several comparison groups in the same study, we halved the number of participants in the shared intervention group, which corrected the error introduced by double-counting [22].

### 2.6. Heterogeneity and Sensitivity Analysis

Statistical heterogeneity was examined graphically by forest plots, standardized Chi-squared (*χ*^2^) test, and *I*^2^ statistic. *I*^2^ statistics were interpreted as follows: statistical significance was considered at a *P* value of <0.05 and an *I*^2^ of >50%. An *I*^2^ of ≥50% might be considered as substantial heterogeneity. Sensitivity analysis was performed to test the influence of each study by visual evaluation of the funnel plot and exclusion sensitivity plot, searching for any asymmetry. Subgroup analyses were conducted according to different control groups: WL/UC, AT, and CBT + Control vs. Control group.

## 3. Results

### 3.1. Searched Studies and Characteristics

**Figure 1:**
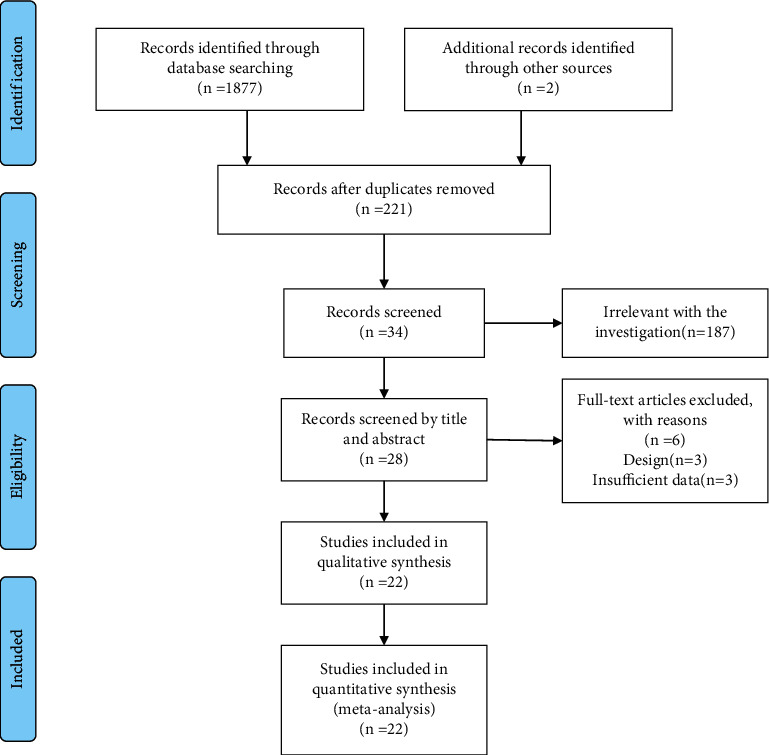
An illustration of the flow at each stage of the study.

The literature search identified 1752 articles from 5 electronic databases. After excluding irrelevant studies, 22 eligible articles, covering 20 separate RCTs, met the inclusion criteria [23–44] (Figure 1). Turner et al. [42] and Cherkin et al. 2016 belong to the same RCT and Harris et al. [30] was part of a larger randomized controlled multicenter trial conducted by Reme et al. [37]. All of the included 20 RCTs were published in English and conducted from nine different countries (6 in the United States, 3 in Germany, 3 in the United Kingdom, 2 in Norway, 2 in Australia, one each in the Netherlands, Sweden, Italy, and Pakistan). In total, 3003 patients were examined. Study sample sizes ranged from 44 to 363 (mean = 152). The intervention types in 15 studies were face-to-face, three studies were Internet-based, and 4 studies were based on telephone, text, audiotape, and mixed methods (face-to-face and telephone). The mean intervention duration of CBT was 10 weeks, ranging from 3 to 54 weeks. Five studies had three months follow-up, 6 studies had six months follow-up, and five included one-year follow-up. One article had nine months of follow-up. The comparison group included WL/UC (*n* = 12), AT (*n* = 7). Other detailed descriptions of the characteristics of the included studies are shown in Table 1. The description of CBT and the comparison groups intervention type are shown in Table 2.

### 3.2. Risk of Bias in Included Studies

**Figure 2:**
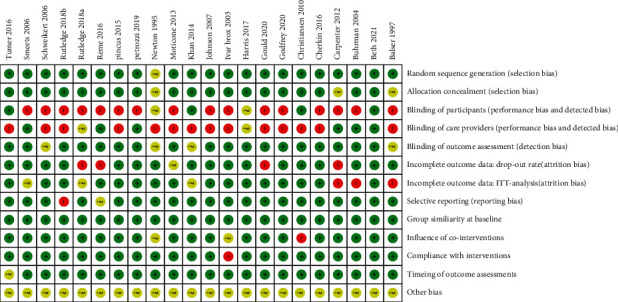
Risk of bias of the included studies. Most studies were low risk in the selection bias, while the performance and detected bias were high risks.

The risk of bias was assessed based on the Cochrane Back and Neck Review Group (Figure 2). All of the 22 studies randomly assigned patients into groups. Random sequence generation was based on computer (*n* = 15) [23, 25, 30–33, 36–43], random number table [24, 27] (*n* = 2), toss dice [26] (*n* = 1), third office [28, 29, 44] (*n* = 3), and unclear randomization method [34] (*n* = 1). Due to the content of CBT intervention, the participants and CBT providers were not blinded in most studies, whereas the outcome accessors were blinded in most studies.

### 3.3. Quality of the Evidence and Effects of Intervention

The quality of the evidence ranged between high and very low due to the performance bias and high heterogeneity. Fifteen studies that investigated pain intensity and 18 studies that investigated disability were included. Five studies investigated the effect of CBT on self-efficacy and five studies assessed the outcome of fear avoidance. The quality of the evidence and the effect estimates of all the outcomes are shown in Table 3.

### 3.4. Primary Outcomes

**Figure 3:**
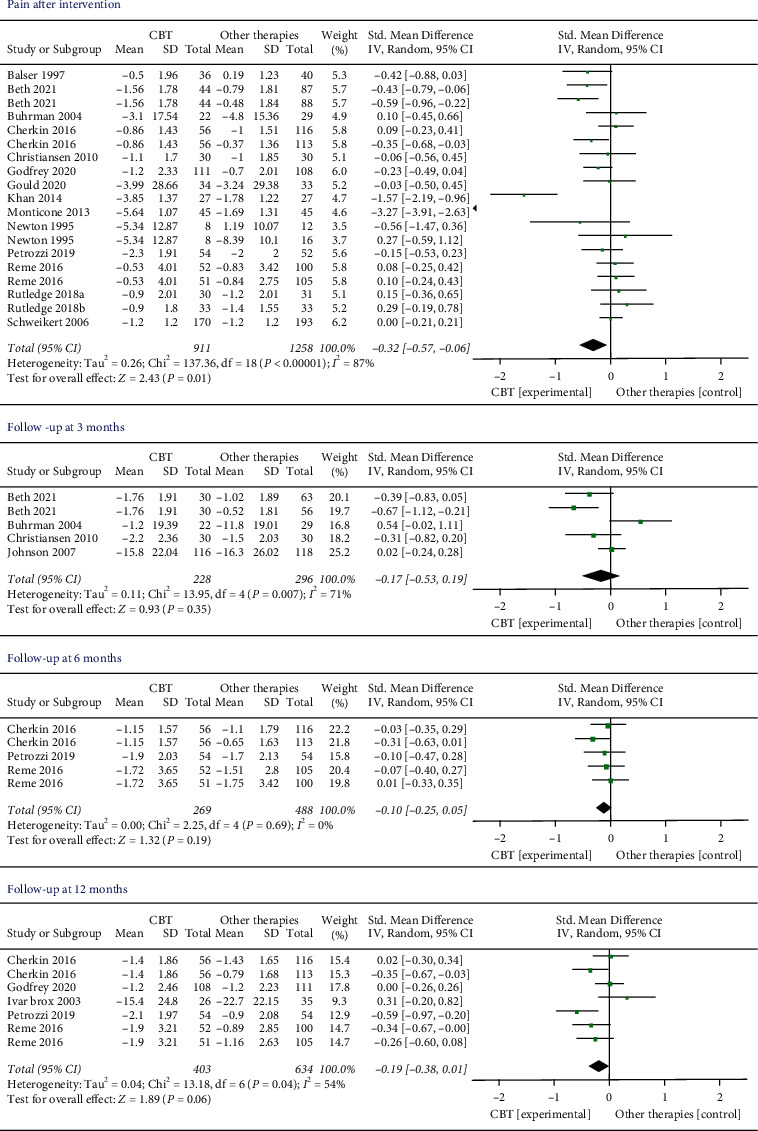
Pain intensity immediately after intervention and during the follow-up period. Compared with other therapies, the overall effect of CBT on pain outcome immediately after intervention was significant (*P* < 0.05). All the follow-up periods failed to show statistical significance.

*Pain Intensity.* Fifteen studies (*n* = 2169 participants) were involved in comparing the effects of CBT with other therapies before and after the interventions. We found low-quality evidence that there was a better effect of CBT on reducing pain compared with other therapies. The effect was statistically significant (SMD −0.32, 95% CI −0.57 to −0.06, *I*^2^ = 87%, *P*=0.01). Two articles (Monticone et al. 2013 [33], Khan et al. 2014 [32]) with extreme outliers and accounted for a large percentage of the statistical heterogeneity were excluded. However, the overall effect changed to a negative result (SMD −0.11, 95% CI −0.23 to −0.01, *I*^2^ = 37%, *P*=0.07). There was no statistical significance at the follow-up 3, 6, and 12 months (Figure 3).

**Figure 4:**
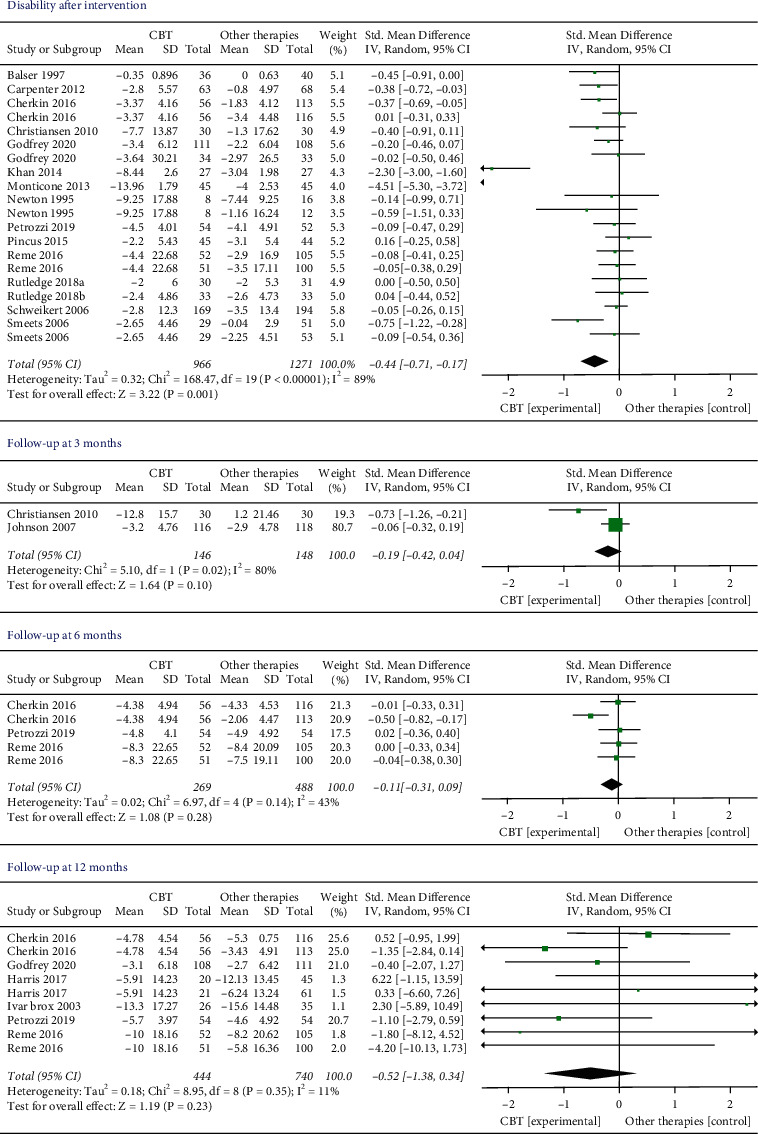
Disability levels immediately after intervention and during the follow-up period. Compared with other therapies, the overall effect of CBT on disability outcome immediately after intervention was significant (*P* < 0.05). All the follow-up periods failed to show statistical significance.

*Disability*. There were 16 trials (*n* = 2237 participants) that compared the effects of CBT to other therapies before and after the interventions. The results showed that CBT provided a significant disability improvement compared with other therapies (SMD −0.44, 95% CI −0.71 to −0.17, *I*^2^ = 89%, *P*=0.001). Two articles [32, 33] with extreme outliers and accounted for a large percentage of the statistical heterogeneity were excluded, while the overall effect remained unchanged (SMD −0.16, 95% CI −0.25 to −0.06, *I*^2^ = 11%, *P*=0.001). Two, 3, and 5 studies measured the pain at 3, 6, and 12 months later, respectively. However, there was no significant difference in all the follow-up times (Figure 4).

### 3.5. Secondary Outcomes

*Self-Efficacy.* Five studies included 1060 participants that assessed the results of self-efficacy. We found low-quality evidence that CBT was significantly more effective than other interventions (SMD 0.27, 95% CI 0.15 to 0.40, *I*^2^ = 74%, *P*=0.0008).

**Figure 5:**
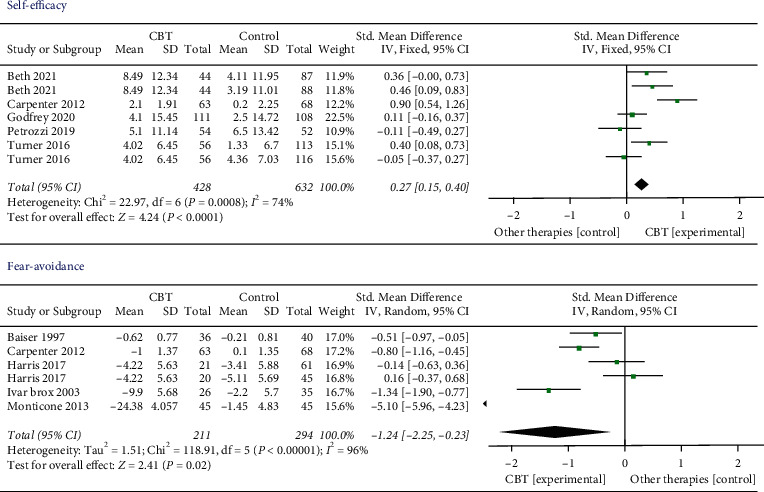
Self-efficacy and fear avoidance after intervention. The overall effect of CBT on self-efficacy and fear avoidance after intervention was in favor of other therapies (*P* < 0.05).

*Fear Avoidance*. Five studies containing 505 participants measured the outcome of fear avoidance. There was low-quality evidence that CBT may produce better effects compared to other interventions. The effect was statistically significant (SMD −1.24, 95% CI −2.25 to −0.23, *I*^2^ = 96%, *P*=0.002) (Figure 5).

### 3.6. Subgroup Analysis

**Figure 6:**
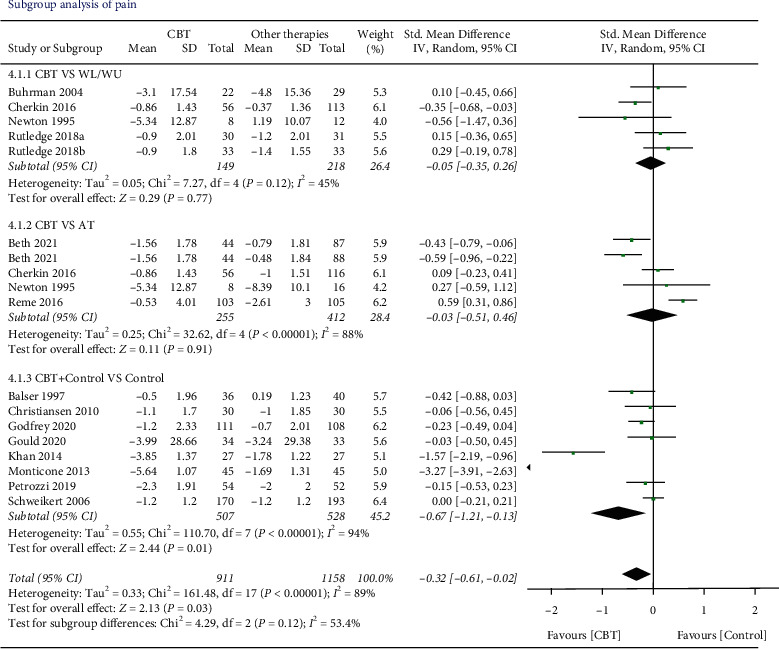
The outcome of the pain of different control subgroups. Combined CBT with other therapies showed a greater overall effect than other therapies alone (*P* < 0.05).

**Figure 7:**
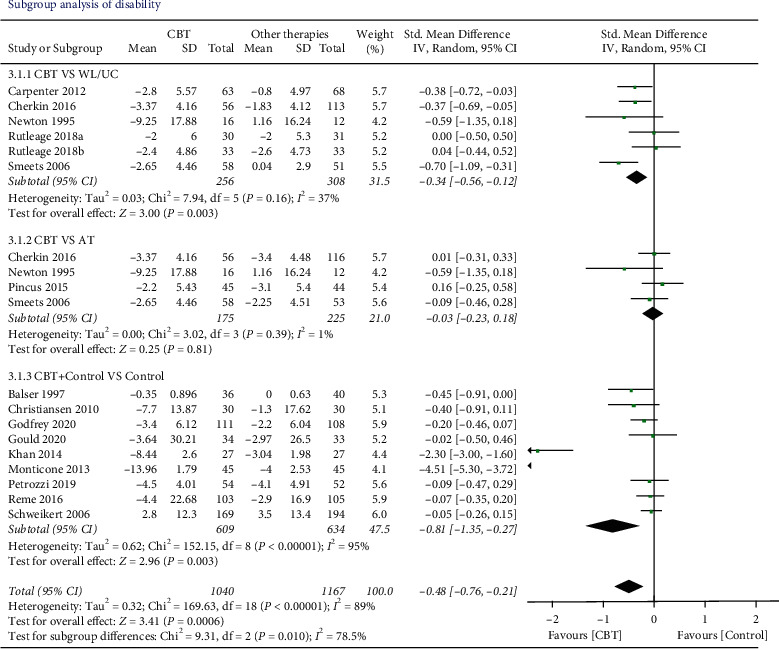
The outcome of disability of different control subgroups. Compared with the waiting list or usual care, the overall effect of CBT on improving disability showed statistical significance (*P* < 0.05). Combining CBT with other therapies showed a greater overall effect than other therapies alone (*P* < 0.05).

We evaluated the subgroup of CBT on posttreatment according to CBT versus control groups (waiting list/usual care, active therapy) and concurrent CBT versus CBT alone about the outcome of pain and disability (Figures 6 and 7). *CBT vs. WL/UC*. Five studies and six studies compared the effects of CBT with WL/UC on the results of pain and disability, respectively. For the result on pain, low-quality evidence suggested that CBT did not result in a statistically better effect than the sham WL/UC group (SMD −0.05, 95% CI −0.35 to 0.26; participants = 367; *I*^2^ = 45%). For disability, moderate-quality evidence suggested that CBT had a significantly better effect than WL/UC (SMD −0.34, 95% CI −0.56 to −0.12; participants = 564; *I*^2^ = 37%, *P*=0.003).

*CBT versus AT*. Four studies examined the effects of CBT versus AT on the result of pain and disability, respectively. It was found that there was no better treatment effect comparing CBT to AT on either pain (SMD −0.03, 95% CI −0.51 to 0.46; participants = 667; studies = 4; *I*^2^ = 88%, *P*=0.91) or disability (SMD 0.03, 95% CI −0.23 to 0.18; participants = 400; studies = 4; *I*^2^ = 1%, *P*=0.81).

*CBT plus Control vs. Control*. For the outcome of pain, eight studies were included in comparing the effects of combined CBT and control with the control alone. The results showed CBT plus control provided a better effect on pain-relieving than control alone (SMD −0.67, 95% CI −1.21 to −0.13; participants = 1035; studies = 8; *I*^2^ = 94%, *P*=0.01), and 9 studies included the outcome of disability within the design of CBT plus control versus control. Statistically significant better effects were found (SMD −0.81, 95% CI −1.35 to −0.27; participants = 1243; *I*^2^ = 95%; *P*=0.003). However, when two articles [32, 33] with extreme outliers were excluded, the overall effect of pain changed to a negative result (SMD −0.12, 95% CI −0.25 to 0.02, *I*^2^ = 0%, *P*=0.09) and the effect of disability remained unchanged (SMD −0.13, 95% CI −0.25 to −0.01, *I*^2^ = 0%, *P*=0.03).

### 3.7. Heterogeneity Inspection and Sensitivity Analysis

By visual inspection, outliers were removed to assess their influence on the overall effect. Two articles [32, 33] included in the analysis showed extreme outliers and raised the heterogeneity to very high values. For Monticone et al. [33], it had no overlap with any other study in the analysis. High efficacy estimate was suspected as the long intervention period (1 year) compared with other studies (mean = 7.95 weeks). However, the author did not offer any explanation in the article. For Khan et al. [32], the author did not provide an explanation for the presence of extreme outliers.

## 4. Discussion

This is the first meta-analysis to evaluate the effect of CBT beyond the intervention period by not restricting CBT intervention types or CBT providers. This is also the first review to investigate the effect of CBT on improving self-efficacy. Twenty-one studies were included in this meta-analysis. Most of the studies were low- to moderate-quality evidence and two studies were found to be of high-quality evidence.

### 4.1. Pain and Disability during Different Periods

For pain intensity immediately after the intervention, 15 studies of low-quality evidence supported the small or very small benefits of CBT over other therapies for reducing pain. However, this finding must be interpreted with caution as there were two outlier articles with high heterogeneity that result in contradicting results upon removal of those two studies. Sixteen studies of low quality of evidence reported CBT was superior to other therapies immediately after intervention to reduce disability. The quality of evidence was hampered by the high heterogeneity and performance bias. Thus, further well-designed RCTs are required to provide high-quality evidence.

No significant difference was observed between CBT and other therapies for relieving pain or improving disability during all follow-up time points. These results seem to suggest that the effects of CBT do not go beyond the intervention period. It is unclear as to the exact reason for the lack of benefit during the follow-up period. It has long been documented that relatively little is known about the specific biobehavioral mechanisms of CBT that lead to chronic pain and disability improvement [45, 46]. Further study is required to establish the optimal strategy to maintain the medium-long-term efficacy of CBT. A systematic review on CBT also indicated that even the short-term effect of CBT is limited and an adequate reinforcement phase is essential to maintain the benefit of CBT acquired during the intervention period [47]. Despite the finding of the present study, further investigation is required to confirm the finding on the benefit of CBT of pain and disability beyond the intervention period as the studies included in this analysis had inadequate follow-up periods.

### 4.2. Secondary Outcomes and Subgroup Analysis

Subgroup analysis indicated significant improvement in pain and disability when CBT is provided in conjunction with other therapies. Compared to alternative active treatments, the intervention regime that incorporated CBT as an adjunct produced significant improvements in the domains of the pain experience, cognitive coping, appraisal (positive coping measures), and reduced behavioral expression of pain [48, 49]. This is among the first review to measure the effect of CBT on improving self-efficacy. Moderate evidence for the improvement of self-efficacy suggested CBT had better effects compared with other therapies. Three out of the five included studies showed significant self-efficacy improvements associated with CBT [27, 42, 43], but the high heterogeneity of the studies prevented quantitative comparisons. Self-efficacy has been highlighted to influence the improvement of pain intensity and functioning. Pre- to posttreatment changes in self-efficacy for managing pain mediated the effects of CBT on pain [50]. However, the effect of CBT on self-efficacy has not been studied adequately and further studies are recommended.

Five studies analyzed the fear avoidance outcome in this review and the pooled effects suggested that CBT could reduce fear avoidance. In patients with CLBP, regression analysis showed that fear avoidance beliefs about work accounted for 23% of the variance of disability in activities of daily living and 26% of the variance of work loss, even after allowing for the severity of pain [51]. A similar review showed that a decrease in avoidance values during treatment was associated with less pain and disability [52]. Therefore, early CBT treatment may promote the recovery of pain and disability.

Considering the potential influence of sociodemographic characteristics, we also investigated the pooled effects from the aspects of educational background, gender, and marital status (see Supplementary materials 2. Subgroup analysis of pain and disability from sociodemographic characteristics: sFig. 1, sFig. 2). The results showed that educational attainment may play a role in CBT intervention outcomes. Participants with higher educational background (college grade or higher) may benefit from the CBT intervention in pain relief (*P*=0.04). One published responder analysis in 2021 also showed some predictors for the treatment effects of yoga, physical therapy, and self-care book, including having higher school education, income, employment, few work-related fear avoidance beliefs, and high pain self-efficacy [53]. One article analyzed the factors that might negatively affect the outcome of CBT in patients with low back pain. It was reported that CLBP patients with anxiety, strong focus on pain, and high medical dependency may be categorized to a nonadaptation group for CBT [54].

### 4.3. Agreements and Disagreements with Other Studies or Reviews

The findings of the primary outcomes for CBT were in line with previous systematic reviews [14, 15] which indicated a greater effect on reducing pain disability. Another review included 10 RCTs that showed CBT + PT was advantageous for reducing pain and disability and enhancing functional capacity in CLBP patients [13], which was consistent with our subgroup conclusion that CBT combined with other therapies was superior to other therapies alone in improving pain and disability. For the outcome of fear avoidance, a study conducted by Baez et al. [55] reported similar findings to the present study. The authors concluded that there was inconsistent, patient-oriented evidence (grade B) to support the use of CBT and psychoeducation to treat fear avoidance beliefs in patients with acute, subacute, and chronic low back pain. However, this review did not give a specific analysis of each type of back. The findings of this study about the follow-up effects of CBT on pain and disability were inconsistent with the findings reported in another meta-analysis conducted by Richmond et al. [16]. The study reported a moderate to large significant effect in favor of CBT compared with active treatment at short- (6–12 weeks) and long-term (26–52 weeks) follow-up. This inconsistency may be explained by different inclusion criteria between the two studies. We included patients diagnosed with chronic low back pain (pain duration >3 months), whereas Richmond et al. included LBP patients at both chronic and subacute (< 6 weeks) phases. Secondly, we analyzed pain and disability outcomes at different follow-up time points which compared the CBT with any control therapies. Richmond et al. [16] divided the comparison group into WL/UC and guideline-based active treatments. Therefore, the included RCTs to analyze were different. The most recent review investigated the effects of preoperative CBT on patients who were scheduled to undergo spine surgery for a degenerative disorder of the lumbar spine. The results showed that there were no additional effects of CBT interventions on outcomes in patients scheduled for lumbar surgery compared to usual care. This may be caused by the included patients at high risk for poor postoperative outcomes [56].

### 4.4. Clinical Significance

The minimal clinical significance will be considered if the pain severity was reported to be a 30% reduction from baseline [57]. Ten of the included studies indicated CBT induced a clinically significant reduction in pain intensity or disability level [23, 24, 28, 29, 32–34, 39, 43, 44], whereas 7 studies reported limited or no clinical effects [25, 30, 31, 35, 38, 40, 41]. Five studies did not report the clinical significance of CBT [26, 27, 36, 37, 42]. Despite the pooled significant effects on reduced disability, improved fear avoidance, and self-efficacy, the results must be interpreted with caution since the effect was estimated by the pooled SMD. There was also a lack of consistency in the implementation of CBT regimes and control therapies. Therefore, no firm conclusion could be drawn on the clinical significance of the observed effect.

### 4.5. Limitations

One of the limitations is the variations and differences between the deliveries of CBT intervention protocol of the included studies which might influence the outcome. CBT is a tailored intervention and the exact protocol, such as intervention delivery format, duration time, and the professional qualification of the providers, may vary between studies. The inherent inability to blind participants to the treatment received was a source of potential performance bias favoring CBT intervention. These substantial differences in the CBT program are particularly problematic for the direct comparison between different studies. Another limitation is that the present review only included research studies published in English which may cause language bias.

## 5. Conclusion

CBT intervention may be beneficial in reducing pain and disability in people with chronic low back pain. CBT as an adjunct to other types of therapy may be more effective than CBT or other therapies alone in reducing pain and disability. CBT may be effective in improving fear avoidance and self-efficacy. Further study is recommended to investigate the long-term benefit of CBT to enable the development of an appropriate strategy to maintain the benefits.

## Figures and Tables

**Table 1:** Basic characteristics of the included studies.

Author year	Country	Total sample size (male/female)	Educational attainment (primary/high)	Marital status (married/unmarried)	Outcomes	Follow-up time (month)
Balser 1997	German	76 (18/58)	NA	NA	Pain intensity, disability^1^	3
Beth 2021	USA	263 (130/131)	6/257	160/103	VAS, PESQ	3
Buhrman 2004	Sweden	51 (19/32)	24/32	NA	Pain diary	3
Carpenter 2012	USA	131 (22/109)	60/71	NA	Pain intensity, RMD, FABQ, self-efficacy scale	
Cherkin 2016	USA	341 (224/117)	26/3155	249/92	Characteristic pain intensity, RDQ (modified)	6, 12
Christiansen 2010	German	60 (23/37)	48/12	36/24	ADL, NRS	3
Godfrey 2020	UK	219 (89/130)	111/134	127/92	NRS, RMD, PESQ	12
Gould 2020	USA	67 (60/7)	NA	NA	Pain intensity^2^, RMDQ	
Harris 2017	Norway	147 (73/74)	87/60	102/45	ODI, FABQ	
Ivar brox 2003	Norway	61 (25/36)	NA	54/7	ODI, FABQ, VAS	
Johnson 2007	UK	234 (94/140)	NA	171/63	VAS, RMDQ	3, 9, 15
Khan 2014	Pakistan	54 (25/29)	NA	NA	VAS, RMDQ	
Monticone 2013	Italy	90 (38/52)	82/8	60/30	NRS, RMD, TSK	
Newton 1995	Australia	44 (NA)	NA	NA	Pain diary, PDI	
Petrozzi 2019	Australia	106 (52/54)	NA	NA	PSEQ, RMDQ, NRS	6, 12
Pincus 2015	UK	99 (35/64)	NA	NA	RMDQ, NRS	6
Reme 2016	Norway	308 (140/168)	190/88	215/93	ODI	6, 12
Rutledge 2018^a^	US	61 (55/6)	25/36	40/21	RMDQ, NRS	
Rutledge 2018^b^	US	66 (41/25)	10/56	40/26	RMDQ, NRS	
Schweikert 2006	Germany	363 (339/24)	NA	NA	Pain, Disability^3^	6
Smeets 2006	Netherlands	162 (80/82)	104/58	NA	VAS, RDQ	
Turner 2016	USA	341 (224/171)	26/315	249/92	PESQ	6, 12

NA: not answer; DDS: Du¨sseldorf disability scale; HCS: Heidelberg coping scale; ^1^pain diary, ^2^descriptor differential scale (higher scores indicating higher pain intensity); ^3^pain (German school grades). Disability: Hannover functional questionnaire. Primary educational attainment, ≤12 years education. High educational attainment, college or higher educational experience.

**Table 2:** CBT and comparison group information.

Author year	Groups	Type of CBT	CBT sessions	CBT duration (W)	Comparison type
Balser 1997	CBT + UC vs. UC	Face-to-face	12 sessions *∗* 120 min	12	UC: various forms of medical treatment such as pain medication, nerve blocks, transcutaneous electrical stimulation, and physical therapy, but not surgery.

Beth 2021	CBT vs. PE CBT vs. ER	Face-to-face	8 sessions *∗* 120 min	8	PE: pain education, matched to empowered relief on 4 key factors: duration, structure, format, and site. ER: empowered relief consists of a single-session, 2-hour pain class that includes pain neuroscience education, mindfulness principles, and CBT skills.

Buhrman 2004	CBT vs. WL	Internet-based	Webpages	8	WL: nonspecific treatment control had been used instead of a waiting list.

Carpenter 2012	CBT vs. WL	Internet-based	Webpages	3	WL: nonspecific treatment control had been used instead of a waiting list.

Cherkin 2016	CBT vs. UC, CBT vs. MBSR	Face-to-face	2 sessions *∗* 120 min *∗* 8 W	8	UC: free to seek whatever treatment but no MBSR training or CBT. MBSR: mindfulness-based stress reduction, a program does not focus specifically on a particular condition such as pain.

Christiansen 2010	CBT + UC vs. UC	Face-to-face	2 sessions *∗* 30 min	3	UC: standard outpatient back pain program.

Godfrey 2020	ACT + PT vs. PT	Face-to-face and telephone call	3 sessions *∗* (60 min *∗* 2 + 20 min)	6	PT: usual physical therapy including manual therapy techniques.

Gould 2020	CBT + placebo vs. placebo	Face-to-face	6 sessions (1 *∗* 90 min + 5 *∗* 60 min)	8	Placebo: a dose of benztropine mesylate 0.125 mg daily was chosen.

Harris 2017	BI vs. BI + CBT vs. BI + PE	Face-to-face	7 sessions *∗* 90 min	12	BI: brief intervention, a brief cognitive, clinical examination program addressing pain and fear avoidance. PE: Group physical exercise consisted of strength and endurance training and relaxation.

Ivar brox 2003	Surgery vs. CBT + PT	Face-to-face	NA	12	Surgery: posterolateral fusion with transpedicular screws of the L4–L5 segment and/or the L5–S1 segment. PT: customarily prescribed physiotherapy, including exercises.

Johnson 2007	CBT + UC vs. UC	Face-to-face and leaflet	8 sessions *∗* 120 min	6	UC: received no further intervention and continued to be treated as usual.

Khan 2014	CBT + exercise vs. exercise	Face-to-face	3 sessions *∗* 12 *∗* ? min	12	Exercise: general exercise protocol under the supervision of a physical therapist.

Monticone 2013	CBT + exercise vs. exercise	Face-to-face	5 *∗* 60 min + 12 *∗* 60 min	54	Exercise: general exercise protocol under the supervision of a physical therapist.

Newton 1995	CBT vs. EMGBF vs. WL	Face-to-face	5 *∗* 90 min	4	EMGBF: electromyographic biofeedback, consisted firstly of a psychoeducational session, then introduced to the pain-tension-pain cycle. WL: nonspecific treatment control had been used instead of a waiting list.

Petrozzi 2019	CBT + PT vs. PT	Internet-based	5 modules, online-based	8	PT: included manual therapy in combination with other modalities such as advice, education, and exercise.

Pincus 2015	CBT vs. PT	Face-to-face	8 sessions *∗* 50 min	12	PT: physiotherapy was delivered as usual within services, with the stipulation that it included at least 60% exercise.

Reme 2016	BI + CBT vs. BI	Audiotaped	7 sessions *∗* ? min	8	BI: brief intervention, a brief cognitive, clinical examination program based on a noninjury model addressing pain and fear avoidance, where return to normal activity and work is the main goal.

Rutledge 2018^a^	CBT vs. UC	Text-based	12 sessions (1 *∗* 120 min + 11 *∗* 30 min)	8	UC: controlled for nonspecific benefits of therapy.

Rutledge 2018^b^	CBT vs. UC	Telephone-based	12 sessions (1 *∗* 120 min + 11 *∗* 30 min)	8	UC: controlled for nonspecific benefits of therapy.

Schweikert 2006	CBT + UC vs. UC	Face-to-face	6 sessions *∗* 90 min	3	UC: standardized conventional 3-week inpatient rehabilitation program consisting of daily physiotherapy, massage of the spinal region, electrotherapeutical measures, 1-hour seminar regarding back training, twice-daily exercise program.

Smeets 2006	CBT vs. PT, CBT vs. WL	Face-to-face	18 sessions, total: 11.5 *∗* 60 min	10	PT: aerobic training on a bicycle and strength and endurance training. WL: not allowed to participate in diagnostic or therapeutic procedures because of their CLBP.

Turner 2016	CBT vs. UC, CBT vs. MBSR	Face-to-face	2 sessions *∗* 120 min *∗* 8 W	8	UC: free to seek whatever treatment but no MBSR training or CBT. MBSR: mindfulness-based stress reduction: a program does not focus specifically on a particular condition such as pain.

UC: usual care; PE: pain education; ER: empowered relief; WL: waiting-list; PT: physical therapy; BI: brief intervention; MBSR: mindfulness-based stress reduction; EMGBF: electromyographic biofeedback.

**Table 3:** Grade of evidence and effect estimates.

Analyses	No of studies and participants	Effect estimates (95% CI)	*I* ^2^ (%)	Grade
Primary outcomes				
Pain				
After intervention	2169 (15 studies)	−0.32 (−0.57 to –0.06)	87	Low^1,2^
3 months follow-up	524 (4 studies)	0.17 (−0.53 to 0.19)	71	Low^1,2^
6 months follow-up	757 (3 studies)	−0.1 (−0.25 to 0.05)	0	High^3^
12 months follow-up	1037 (5 studies)	−0.19 (−0.38 to 0.01)	54	Low^1,2^
Disability				
After intervention	2237 (16 studies)	−0.44 (−0.71 to −0.17)	89	Low^1,2^
3 months follow-up	294 (2 studies)	−0.19 (−0.42 to 0.04)	80	Very low^1,2,3^
6 months follow-up	757 (3 studies)	−0.11 (−0.31 to 0.09)	43	High
12 months follow-up	1184 (6 studies)	−0.52 (−1.38 to 0.34)	11	Moderate^1^
Secondary outcomes				
Fear avoidance	505 (5 studies)	−1.24 (−2.25 to −0.23)	96	Low^1,2^
Self-efficacy	1060 (5 studies)	0.27 (0.15 to 0.40)	74	Moderate^1^
Subgroups analyses				
Pain				
CBT vs. WL/UC	367 (5 studies)	−0.05 (−0.35 to 0.26)	45	Low^1,3^
CBT vs. AT	667 (4 studies)	−0.03 (−0.51 to 0.46)	88	Moderate^2^
Concurrent CBT	1035 (8 studies)	−0.67 (−1.21 to −0.13)	94	low^1,2^
Disability				
CBT vs. WL/UC	564 (6 studies)	−0.34 (−0.56 to −0.12)	37	Moderate^1^
CBT vs. AT	400 (4 studies)	−0.03 (−0.23 to 0.18)	1	Moderate^1^
Concurrent CBT	1243 (9 studies) (9 studies)	−0.81 (−1.35 to −0.27)	95	Moderate^2^

GRADE interpretation: ^1^>50% of subjects came from studies with a performance bias; ^2^the heterogeneity was large (*I*^2^ >50%, representing potentially substantial heterogeneity); ^3^the total population size is less than 400 or there is only one study.

## Data Availability

The data of primary and secondary results supporting this meta-analysis are from previously reported studies and datasets, which have been cited.

## Supplementary Materials

Supplementary Materials Supplementary Material 1: PRISMA Checklist. Supplementary Material 2: subgroup analysis of pain and disability from sociodemographic characteristics: sFig.1, sFig.2.
