# Supplementary material for: Evaluation of Cognitive Behavioral Therapy on Improving Pain, Fear Avoidance, and Self-Efficacy in Patients with Chronic Low Back Pain: A Systematic Review and Meta-Analysis
Source: Pain Res Manag. 2022 Mar 19;2022:4276175. doi: 10.1155/2022/4276175 (PMC8957446; doi:10.1155/2022/4276175)
Supplement: Supplementary Materials — Supplementary Material 1: PRISMA Checklist. Supplementary Material 2: subgroup analysis of pain and disability from sociodemographic characteristics: sFig.1, sFig.2. [file 4276175.f1.zip › 4276175.f1/Supplementary Material 2_Subgroup analysis of pain and disability from sociodemographic characteristics.docx]

Subgroup analysis of pain and disability from sociodemographic characteristics.


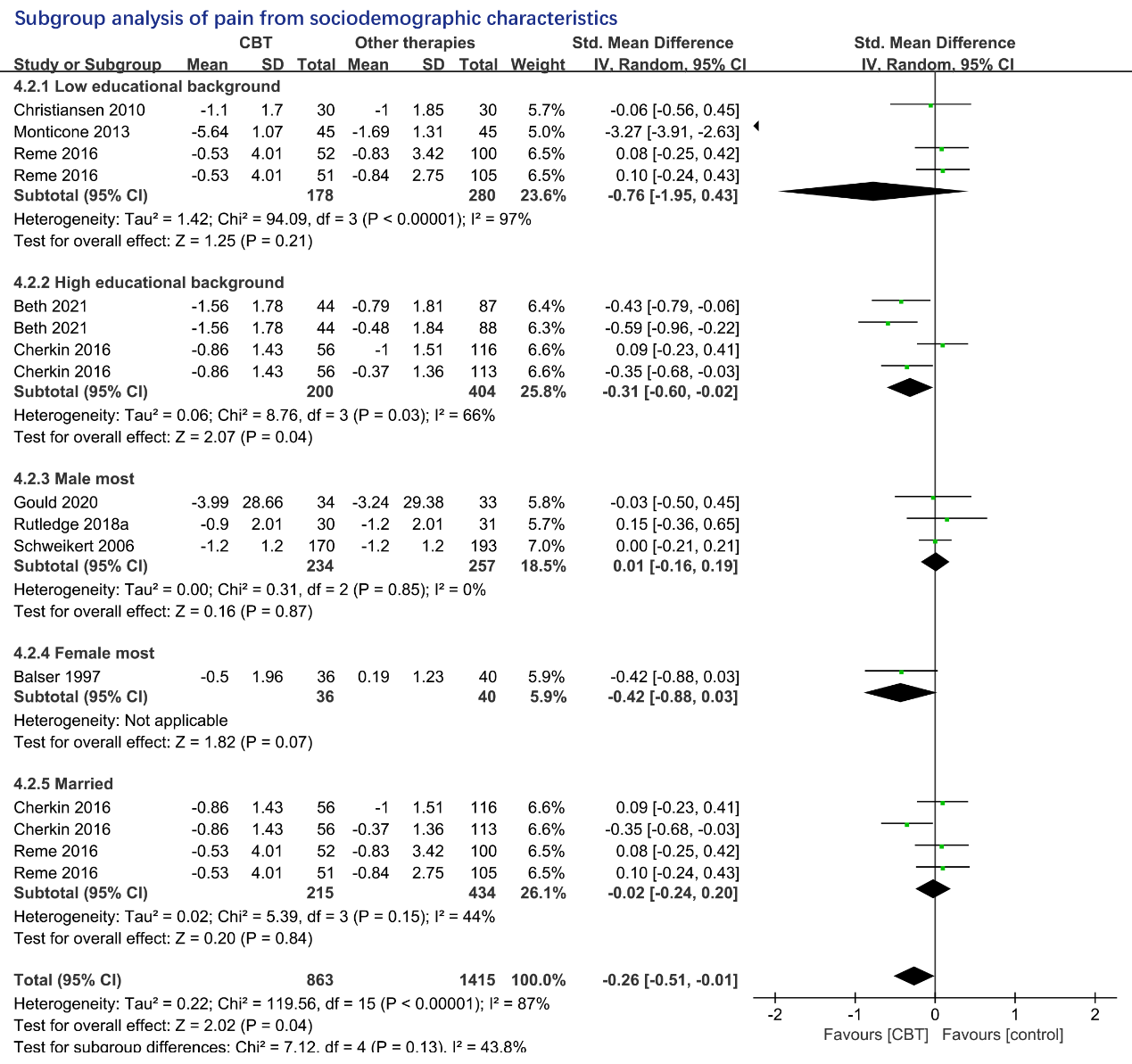


Figure 1. The outcome of pain of different sociodemographic characteristics subgroups.


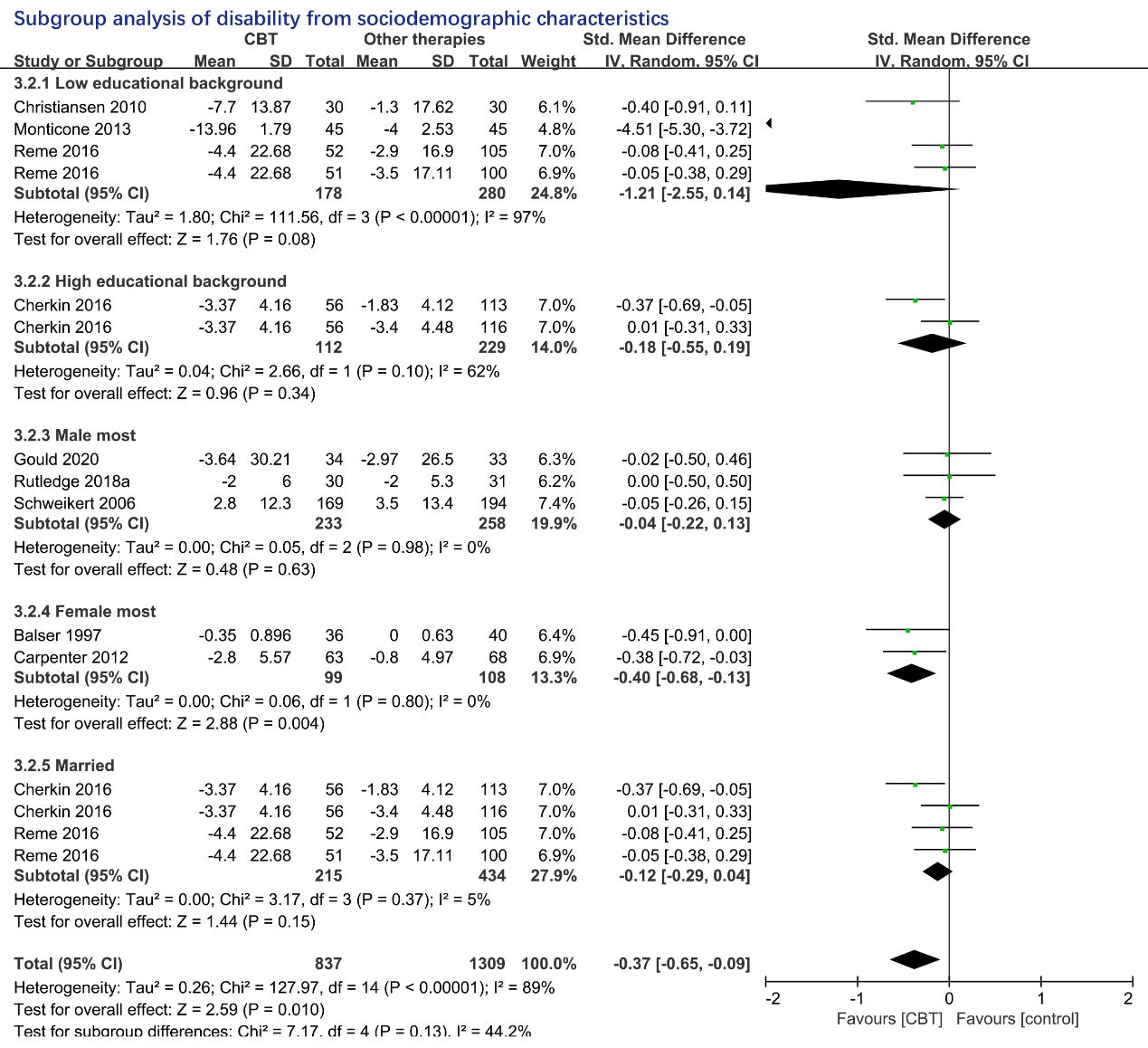


Figure 2. The outcome of disability of different sociodemographic characteristics subgroups.
